# Identification of the Benignity and Malignancy of BI-RADS 4 Breast Lesions Based on a Combined Quantitative Model of Dynamic Contrast-Enhanced MRI and Intravoxel Incoherent Motion

**DOI:** 10.3390/tomography8060223

**Published:** 2022-10-31

**Authors:** Wenjuan Xu, Bingjie Zheng, Hailiang Li

**Affiliations:** 1 Academy of Medical Science, Zhengzhou University, Zhengzhou 450001, China; 2 Department of Radiology, The Affiliated Cancer Hospital of Zhengzhou University, Zhengzhou University, Zhengzhou 450008, China

**Keywords:** breast lesion, BI-RADS, dynamic contrast–enhanced magnetic resonance imaging, intravoxel incoherent motion, diffusion weighted imaging

## Abstract

The aim of this study was to explore whether intravoxel incoherent motion (IVIM) combined with a dynamic contrast–enhanced magnetic resonance imaging (DCE–MRI) quantitative model can improve the ability to distinguish between benign and malignant BI-RADS 4 breast lesions. We enrolled 100 patients who underwent breast MRI at our institution and extracted the quantitative parameters of lesions with a post-processing workstation. Statistical differences in these parameters between benign and malignant BI-RADS 4 lesions were assessed using a two independent samples t-test or a Mann–Whitney U test. Binary logistic regression analysis was performed to establish five diagnostic models (model__ADC_, model__IVIM_, model__DCE_, model__DCE+ADC_, and model__DCE+IVIM_). Receiver operating characteristic (ROC) curves, leave-one-out cross-validation, and the Delong test were used to assess and compare the diagnostic performance of these models. The model__DCE+IVIM_ showed the highest area under the curve (AUC) of 0.903 (95% confidence interval (CI): 0.828–0.953, sensitivity: 87.50%, specificity: 85.00%), which was significantly higher than that of model__ADC_ (*p* = 0.014) and model__IVIM_ (*p* = 0.033). The model__ADC_ had the lowest diagnostic performance (AUC = 0.768, 95%CI: 0.672–0.846) but was not significantly different from model__IVIM_ (*p* = 0.168). The united quantitative model with DCE–MRI and IVIM could improve the ability to evaluate the malignancy in BI-RADS 4 lesions, and unnecessary breast biopsies may be obviated.

## 1. Introduction

In 2021, breast cancer has surpassed lung cancer to be the most common cancer in the world, accounting for a severe global burden, especially among women [1]. The identification of benign and malignant breast lesions is the most fundamental and major step in the treatment of breast diseases. As a sensitive and non-invasive examination technique, magnetic resonance imaging (MRI) plays an important role in the detection and classification of breast cancer, as well as in the observation of changes in treatment, and is widely used in clinical practice.

Breast lesions can be classified into six categories according to Breast Imaging-Reporting and Data System–Magnetic Resonance Imaging (BI-RADS–MRI) [2]. Lesions without typical signs of malignancy but with sufficiently suspicious presentation were classified as BI-RADS 4 (malignancy probability >2% but <95%) [2]. Because of the high likelihood of malignancy, the biopsy of suspicious areas is recommended in all patients with BI-RADS 4 lesions to characterize their pathology [3]. However, the wide range of malignancy possibility has also led to unnecessary histological biopsies in some patients, which is traumatic. Therefore, further non-invasive precise diagnosis of benign and malignant BI-RADS 4 lesions is necessary.

Dynamic contrast–enhanced MRI (DCE–MRI) was proven to be a sensitive breast screening technique [4]. Some studies have investigated the morphological appearance of breast lesions on DCE–MRI, thus providing clues for the formulation of BI-RADS–MRI [5,6]. However, morphological manifestations are subjective, and some benign lesions show similar morphological features with those of breast cancer. In order to improve the ability to distinguish benign and malignant BI-RADS 4 lesions accurately, some researchers tried to explore the diagnostic value of the pharmacokinetic parameters of DCE–MRI. They found that these parameters showed initial value [7,8].

Diffusion weighted imaging (DWI) was considered a reliable adjunct to DCE–MRI, which could help visualize and quantify the random motion of water molecules in tissues, which is influenced by cell density and tissue microstructure, thus creating a contrast in tissue with no injection of contrast agents [9]. The apparent diffusion coefficient (ADC) value calculated by the single exponential model can quantitatively reflect the diffusion of water molecules in tissues. However, microcirculation perfusion and pure molecular diffusion simultaneously contribute to the ADC value, which may hinder its ability to characterize the tissue microstructure [10]. The theory of intravoxel incoherent motion (IVIM), which refers to translational movements that present a distribution of speeds in orientation and/or amplitude within a given voxel and during the measurement time, was first described by Le Bihan et al. It is based on a bi-exponential model to calculate multiple-b-value DWI data, which can simultaneously evaluate tissue diffusivity and tissue microvascular perfusion, providing richer tissue microstructural information [11].

Studies have shown that ADC–DWI and IVIM have potential application value in distinguishing benign and malignant breast lesions [12,13]. Therefore, some researchers tried to compare the diagnostic efficacy of ADC–DWI and IVIM but obtained inconsistent results [14,15]. Xiao et al. believed that the diagnostic efficacy of IVIM was higher than that of ADC–DWI [14], while Weili Ma believed that there was no statistical difference between them [15]. The potential value of combining DWI and DCE–MRI in the differential diagnosis of breast lesions has also been pointed out, but it was usually based on ADC–DWI [16], and a few related studies based on IVIM mainly combined the morphological features of DCE–MRI [17]. To our knowledge, no previous research has attempted to simultaneously assess the value of ADC–DWI, IVIM, quantitative parametric model of DCE–MRI, and their combined models in the diagnosis of BI-RADS 4 breast lesions. Therefore, this study aimed to investigate whether different DWI combined with DCE–MRI quantitative models can improve the ability to identify the malignancy of BI-RADS 4 lesions.

## 2. Materials and Methods

### 2.1. Patient Selection

Based on the following inclusion criteria, we included 100 patients with 100 breast lesions (benign: 20, malignant: 80) who underwent breast MRI at our institution from June 2016 to July 2017 and underwent subsequent treatment. Each patient was categorized according to the fifth edition of the BI-RADS–MRI guidelines. The inclusion criteria were as follows: (1) The patient was diagnosed with BI-RADS 4 breast lesions at MRI. (2) Pathological diagnosis was confirmed by needle biopsy or surgical specimen. (3) The patient had full MRI images and underwent an MRI sequences scan before biopsy. (4) The image quality of the patient met the diagnostic criteria. (5) The patient did not receive radiotherapy, chemotherapy, or surgery in the past. General clinical data and information on patients were retrospectively collected. Histopathological diagnosis of lesions was obtained by the analysis of image-guided biopsies or surgical samples. All pathological findings were defined according to the World Health Organization classification of breast lesions [18]. This study was a retrospective study that was approved by the Institutional Ethics Committee, and informed consent was waived. The study was conducted in accordance with the Declaration of Helsinki.

### 2.2. MRI Image Acquisition

MRI was acquired on a 3.0 T Skyra device (Siemens Healthcare, Erlangen, Germany) with an eight-channel bilateral breast coil in the prone position. All patients underwent breast MRI, including T1-weighted imaging, T2-weighted imaging, DCE–MRI, and transverse multiple-b DWI. DCE–MRI was performed using time-resolved angiography with interleaved stochastic trajectories sequence and the following parameters: the repetition time (TR), 4.18 ms; the echo time (TE), 1.31 ms; field of view (FOV), 640 × 560 mm^2^; slice thickness, 2.0 mm; no gap; matrix, 320 × 249; flip angle, 12°; temporal resolution, 7.84 s/phase; and acquisition time (TA), 5 min and 33 s. At the beginning of the fourth DCE–MRI frame acquisition, an intravenous bolus injection of 0.2 mmol/kg Gd-DTPA-BMA (Omni-Scan, GE Healthcare, Dublin, Ireland) was administered at a rate of 2.5 mL/s, followed by a 20 mL saline flush.

Transverse multiple-b DWI was acquired using an ISHIM sequence before the DCE–MRI. Nine b values were used: 0, 25, 50, 75, 100, 200, 400, 600, 800 s/mm^2^. The corresponding parameters were as follows: TR, 4209 ms; TE, 58 ms; FOV, 640 × 560 mm^2^; slice thickness, 4.0 mm; matrix, 128 × 67; slice-gap, 4.4 mm; flip angle, 90°; bandwidth, 2440 Hz/pixel; 1 average. The total scan time for the multiple-b DWI sequence was 3 min 34 s.

### 2.3. Image Analysis

ADC values of breast lesions were calculated from a single exponential fitting model of signal intensities at b = 0 and b = 800 s/mm^2^:

S_b_ = S_0_·exp^−bADC^ (1)

The pure diffusion coefficient D, the perfusion-related diffusion coefficient D* and the perfusion fraction f were obtained by the following bi-exponential fitting model as described by Le Bihan et al. [19]:

S_b_/S_0_ = (1 − f)·exp^(−b·D)^ + f·exp^(−b(D+D*)^ (2)

where S_b_ represents the signal intensity with a specific b value, and S_0_ represents the signal intensity without a diffusion gradient. Since the contribution of D* to signal attenuation is negligible at high b values (b > 200 s/mm^2^), a single exponential fitting equation (S_b_ = S_0_·exp^−bD^) was used to determine D. Then, with the result D as a fixed parameter, D is applied to the above bi-exponential equation (Equation (2)), and D* and f are derived using all the values of b.

All post-processing operations of DWI images were performed on a workstation (Advantage Workstation 5.0, GE Healthcare, Saint Louis, MO, USA) and were analyzed with FuncTool 9.4.05 MADC and ADC software, respectively, and D, D*, f, ADC parameter maps were obtained. Two radiologists (BZ and WX, with 10 and 2 years of experience in breast image interpretation, respectively), blinded to pathological findings, clinical data, and other imaging findings, reviewed all images and delineated regions of interest (ROIs), respectively. With T2WI and DCE–MRI images as references, they determined the extent of the lesion on the corresponding IVIM and ADC parameter maps and then manually delineated the ROI along the lesion edge at the slice of the maximum lesion diameter. Similar to previous studies [17], areas of apparent cystic lesions, necrosis, calcification, and hemorrhage were avoided. Subsequently, the software automatically calculated the quantitative parameters D, D*, f, ADC within the ROI.

**Figure 1:**
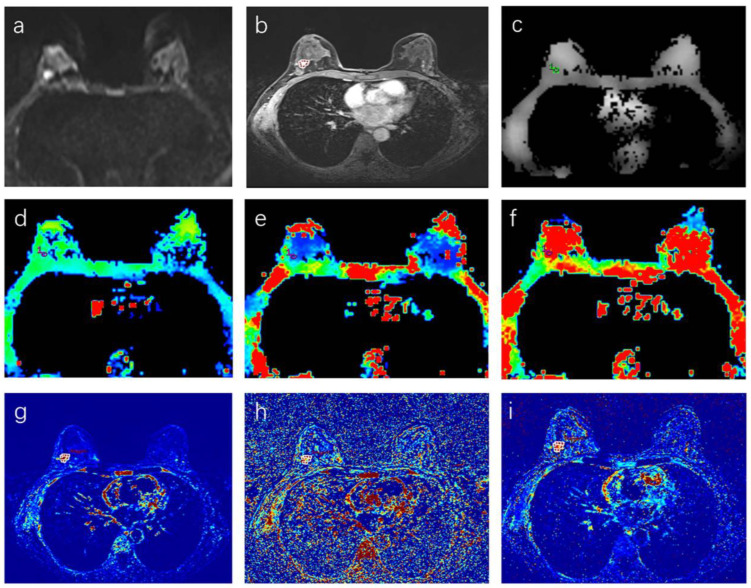
(**a**–**i**) A 46-year-old woman with a malignant BI-RADS 4 lesion in the right breast. (**a**) Diffusion-weighted imaging at b = 800 mm/s^2^. (**b**) Dynamic contrast-enhanced imaging, (**c**) ADC map, (**d**) D map, (**e**) D* map, (**f**) f map, (**g**) K^trans^ map, (**h**) K_ep_ map, (**i**) V_e_ map. The circle stands for the delineated ROI.

The same two radiologists analyzed DCE–MRI images with Omni-Kenetics software. Based on the Extended Tofts Linear mode, the software automatically obtained pharmacokinetic parameter maps. ROIs, which were as consistent as possible with the ROIs on the IVIM and ADC images, were drawn by radiologists at the early enhancement phase (Figure 1). Then the pharmacokinetic parameters (K^trans^, K_ep_, V_e_) of the ROI were calculated automatically by the software.

### 2.4. Statistical Analysis

Statistical analysis was performed on IBM SPSS (v26.0; Chicago, IL, USA), MedCalc (v19.6; Ostend, Belgium) and R (version 4.2.1). The intraclass correlation coefficient (ICC) was used to evalute the agreement of the quantitative parameters measured by the two radiologists. Data were analyzed for normality with the Shapiro–Wilk test, followed by an analysis of variance homogeneity with the Levene test. A two independent samples *t*-test or a Mann–Whitney U test was used to evaluate the statistical differences of quantitative parameters between benign and malignant lesions. Binary logistic regression analysis (Method: Forward: LR) was performed to establish five diagnostic models (model__ADC_, model__IVIM_, model__DCE_, model__DCE+ADC_, and model__DCE+IVIM_) with a variable selection criterion of *p* < 0.05. The receiver operating characteristic (ROC) curves, leave-one-out cross-validation (LOOCV), and the Delong test were used to evaluate and compare the diagnostic performance of these models.

## 3. Results

### 3.1. Patient Characteristics

We screened 176 consecutive patients defined as BI-RADS 4 at MRI, excluding 12 patients without IVIM sequences, 52 patients who underwent needle biopsy before MRI, 5 patients whose tumors were too small to accurately delineate the ROI, and 7 patients with incomplete images. Finally, 100 patients with 100 breast lesions (benign: 20, malignant: 80) were included in this study, and their characteristics are summarized in Table 1. The mean age of all patients was 47.4 years, with a range of 26–73 years. The mean age of patients in the benign lesion group was significantly lower than that in the malignant lesion group (*p* = 0.001).

### 3.2. Consistency Test

After the inter-observer agreement analysis of all parameters, it was found that the ICCs of all parameters was greater than 0.82 except for D*__min_ (ICC = 0.419) and K_ep_min_ (ICC = 0.701), and the details are shown in Table 2, indicating that these parameters measured by the two radiologists were in good agreement. Therefore, parameters in good agreement were subjected to subsequent statistical analysis.

### 3.3. DWI and DCE–MRI Quantitative Parameters in Benign and Malignant Breast Lesions

The D__mean_, D__min_, D__max_, D*__mean_, and D*__max_ values of malignant lesions were significantly lower than those of benign lesions (all *p* < 0.05), while the values of f__mean_ and f__min_ were significantly higher than those of benign lesions (*p* = 0.035, 0.009, respectively). The ADC-related parameter values of benign lesions were significantly higher than those of malignant lesions (all *p* < 0.001), but the DCE-related pharmacokinetic parameters such as K^trans^__max_, K_ep_max_, K_ep_median_, and K_ep_mean_ values were significantly lower than those of malignant lesions (all *p* < 0.05). No significant differences in V_e_-related parameters were observed between benign and malignant lesions. More detailed data are shown in Table 3.

**Figure 2:**
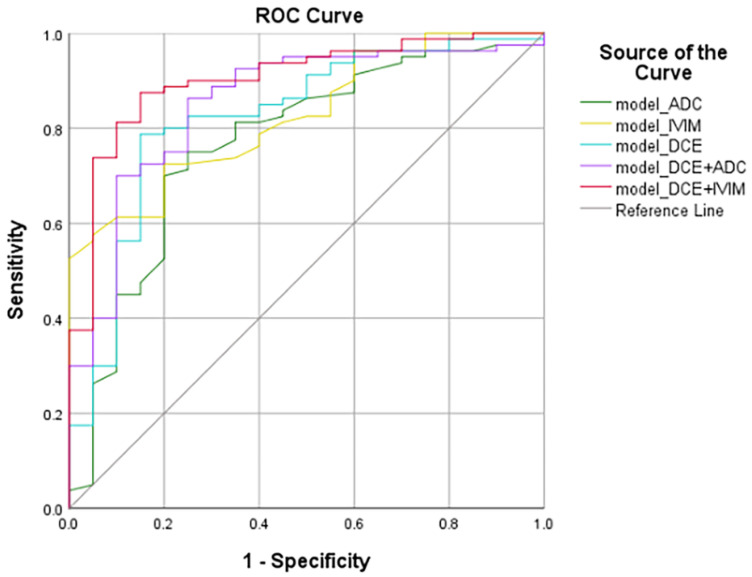
ROC curves of the models. The ROC curves were generated by models based on: ADC–DWI, IVIM, DCE–MRI, DCE–MRI + ADC–DWI, DCE–MRI + IVIM. ROC, receiver operating characteristic.

The ROC curves of the diagnostic performance of each multivariate model are shown in Figure 2. The AUC, 95% CI, standard error, sensitivity, specificity, accuracy, and *p*-values of these models are shown in Table 4. The diagnostic performance of these models was compared by the Delong test, and the results with significant differences are shown in Table 5. Compared with the model__ADC_ (AUC = 0.768, 95%CI: 0.672–0.846), the model__IVIM_ (AUC = 0.826, 95%CI: 0.737–0.894, *p* = 0.168 by Delong test) improved the diagnostic performance of BI-RADS 4 lesions, but the difference was not significant. The diagnostic efficiency of model__DCE+IVIM_ was the highest, with an AUC of 0.903, and the sensitivity and specificity were 87.5% and 85%, respectively.

## 4. Discussion

This study analyzed the role of ADC–DWI, IVIM, and DCE–MRI in identifying the benignity and malignancy of BI-RADS 4 breast lesions. Combining the quantitative variables of these sequences, this study concluded that the single IVIM and the DCE model showed better diagnostic performance compared to the single ADC–DWI model, although the difference was not significant. The combined model of IVIM and DCE–MRI could improve the sensitivity and specificity of diagnosis most and help patients avoid unnecessary breast biopsy.

Breast MRI has the advantages of good soft tissue resolution and no radiation [20,21]. The likelihood of BI-RADS 4 breast lesions being malignant ranged from 2% to 95%, but the actual positive predictive value of breast lesions ranged from 25.7% to 59.2% [22,23,24]. Since MRI is insensitive to microcalcifications, it can easily lead to false-negative diagnoses [25], and morphological assessments are highly subjective, which may lead to the overdiagnosis of patients. Therefore, we combined the quantitative variables of DCE–MRI, IVIM, and ADC–DWI, established multiple quantitative models, and compared their diagnostic performance to provide radiologists and oncologists with a more reliable quantitative assessment tool.

In multivariate logistic regression analysis, ADC__min_, D__mean_, and K_ep_max_ were found to be independent predictors of breast malignancy. ADC values can be used to estimate tumor biological characteristics such as water content, tissue cell density, vessel density, and cell membrane integrity [11]. The ADC__min_ value of malignant lesions was lower than that of benign lesions, which was the same as the previous research results [26]. This might be attributed to the continuous proliferation of tumor cells, the increase in the synthesis of macromolecular substances in the cytoplasm, the release of a large amount of necrotic substances, the reduction of extracellular space, the increase of bound water content, and the restricted diffusion of free water molecules [27]. A meta-analysis showed that the ADC value for distinguishing between benign and malignant breast lesions range from 0.92 to 1.61 × 10^−3^ mm^2^/s [28]. In this study, the optimal threshold for ADC__min_ was 1.31 × 10^−3^ mm^2^/s, which is consistent with previous findings.

D is the diffusion coefficient of pure water in the tissue. Malignant breast lesions are associated with the limited diffusion of water molecules due to rapid proliferation, high density, and the shrinking of the extracellular space of tumor cells. The D* value and the f value mainly reflect the state of blood perfusion. In this study, the values of D-related parameters, D*__mean_, and D*__max_, in malignant lesions were significantly lower than those in benign lesions (all *p* < 0.05), while f__min_ and f__mean_ were significantly higher (*p* = 0.009; *p* = 0.035), which was consistent with the results of Yichuan Ma [29]. However, there were also some different opinions; Nan Meng believed that the D* value of breast cancer was higher than that of benign lesions [30], while Liang et al. believed that the D* value of benign and malignant lesions was not statistically different [31]. The reason for this discrepancy may be patient selection bias: fibroadenoma and inflammation with hyperperfusion in this study accounted for 60% of benign lesions, making D * and f of benign lesions overlap with malignant lesions. In addition, the D* value is affected by age and menstrual status. The D* value of the normal breast tissue of postmenopausal subjects is significantly lower than that of premenopausal subjects, and the premenopausal D* value (low and middle age groups) fluctuates with the menstrual cycle [32]. However, potential influencing factors such as age and menstrual cycle were not considered in this study, which might have a certain impact on the accuracy of the D* value.

DCE–MRI can characterize the complex microcirculation in living tissue and provide quantitative information on vascular permeability and angiogenesis. Higher K^trans^ and K_ep_ values reflect higher microvascular blood flow, vascular density, and vascular permeability in diseased tissue [33]. In this study, the K^trans^__max_ and K_ep_-related parameter values of malignant lesions were significantly higher than those of benign lesions, which was consistent with the results of previous studies [34]. This may be attributed to the increased leakage of contrast agents due to incomplete vascular endothelial cells and high vascular permeability in malignant breast lesions [35]. In addition, local hypoxia and necrotic sites in malignant lesions release angiogenic cytokines, leading to increased angiogenesis and microvascular leakage [36]. The K_ep_max_ value was considered an independent predictor of malignancy in the logistic regression analysis, while the K^trans^ value was not included, possibly because K^trans^ value was potentially affected by conditions such as cardiac output and hypertension that affect blood perfusion [36].

After the Delong test, we found that the diagnostic performance of the model__IVIM_ was higher than that of the model__ADC_, but their differences did not reach statistical significance, which was the same as the findings of Iima M and Baxter GC [37,38], who recommended using ADC–DWI in the clinic to distinguish benign from malignant lesions in order to reduce image acquisition time. However, the conclusion of Xiao et al. was different. They believed that the diagnostic performance of IVIM was significantly higher than ADC–DWI [14]. We speculated that these differences may be due to the study population, the size and number of the b-value, ROI location, and post-processing software. The diagnostic efficiency of the model__DCE+IVIM_ was significantly higher than that of the model__ADC_ and model__IVIM_ and higher than the model__DCE+ADC_ and model__DCE_ but did not reach statistical significance, suggesting that the combination of IVIM and DCE–MRI quantitative parameters could better predict the malignancy of BI-RADS 4 breast lesions, which might be attributed to the combination of assessing microvascular perfusion and cell proliferation. Although the acquisition time of IVIM was longer, making it difficult to apply in clinics, some studies suggested that simplified IVIM could also achieve the same diagnostic effect as conventional IVIM [39]. However, there was no research to compare the diagnostic effect of the simplified model__DCE+IVIM_ with that of the conventional model__DCE+IVIM_. We will try to discuss this in the next step.

There were some limitations of this study: (1) This study was a single-center retrospective study. The sample size of benign lesions was small, and fibroadenomas were the main ones, so there might be sampling bias. (2) In this study, the manual delineation of ROI might have measurement errors. However, in the consistency analysis of this study, it was found that the consistency of manual delineation was good. (3) Factors such as patient age and menstrual cycle were not considered when patients were included in the study. All of the above need to be further studied by increasing the sample size in the follow-up process.

## 5. Conclusions

In conclusion, the model built with DCE–MRI and IVIM quantitative parameters seems to be a more reliable tool for evaluating the malignancy in BI-RADS 4 lesions compared to the single ADC–DWI and IVIM models.

## Figures and Tables

**Table 1:** Basic clinical information of enrolled patients.

Parameter	Number of Patients
Benign leasions	20
Mean age (years)	40.8 (26–61)
Histological restult	
Fibroadenoma	9
Granulomatous mastitis	3
Adenomatosis	3
Phyllodes tumor (benign)	3
Fibrocystic change	2
Malignant leasions	80
Mean age (years)	49.0 (29–73)
Histological restult	
Ductal carcinoma in situ	8
Invasive ductal carcinoma	62
Invasive lobular carcinoma	5
Mucinous carcinoma	3
Paget’s disease	1
Metaplastic carcinoma	1

Data in parentheses are range.

**Table 2:** Consistency test results for each parameter.

Parameter	ICC	Parameter	ICC	Parameter	ICC
K^trans^__min_	0.875	V_e_min_	0.844	D*__min_	0.419
K^trans^__max_	0.865	V_e_max_	0.876	D*__max_	0.867
K^trans^__median_	0.951	V_e_median_	0.991	f__mean_	0.913
K^trans^__mean_	0.960	V_e_mean_	0.981	f__min_	0.865
K_ep_min_	0.701	D__mean_	0.913	f__max_	0.821
K_ep_max_	0.946	D__min_	0.911	ADC__mean_	0.999
K_ep_median_	0.906	D__max_	0.916	ADC__min_	0.997
K_ep_mean_	0.919	D*__mean_	0.954	ADC__max_	0.997

ICC, intraclass correlation coefficient. “D*” is the perfusion-related diffusion coefficient calculated by the post-processing software.

**Table 3:** Comparison of parameters between benign and malignant lesions.

Parameter	Benign Lesions (*n* = 20)	Malignant Lesions (*n* = 80)	*p* Value
D__mean_ (×10^−3^ mm^2^/s)	1.41 ± 0.27	1.07 ± 0.26	**<0.001 ^#^**
D__min_ (×10^−3^ mm^2^/s)	1.21 (1.02,1.60)	0.93 (0.74,1.11)	**<0.001**
D__max_ (×10^−3^ mm^2^/s)	1.51 ± 0.25	1.18 ± 0.26	**<0.001 ^#^**
D*__mean_ (×10^−3^ mm^2^/s)	27.05 (10.98,78.65)	15.20 (8.37,28.05)	**0.032**
D*__min_ (×10^−3^ mm^2^/s)	9.88 (5.70,31.33)	7.97 (4.87,13.38)	0.289
D*__max_ (×10^−3^ mm^2^/s)	44.65 (21.05,29.38)	22.50 (13.20,46.30)	**0.025**
f__mean_ (%)	11.00 (5.17,15.48)	13.70 (11.20,19.70)	**0.035**
f__min_ (%)	7.44 (2.89,11.20)	10.25 (7.94,12.55)	**0.009**
f__max_ (%)	16.90 (9.59,24.48)	17.60 (14.53,29.15)	0.252
ADC__mean_ (×10^−3^ mm^2^/s)	1.52 ± 0.25	1.28 ± 0.25	**0.001 ^#^**
ADC__min_ (×10^−3^ mm^2^/s)	1.46 ± 0.26	1.22 ± 0.25	**0.001 ^#^**
ADC__max_ (×10^−3^ mm^2^/s)	1.58 ± 0.25	1.35 ± 0.25	**0.001 ^#^**
K^trans^__min_ (min^−1^)	0.06 (0.03,0.09)	0.06 (0.03,0.18)	0.558
K^trans^__max_ (min^−1^)	0.42 (0.15,1.71)	1.02 (0.46,2.38)	**0.011**
K^trans^__median_ (min^−1^)	0.19 (0.09,0.60)	0.35 (0.15,0.75)	0.095
K^trans^__mean_ (min^−1^)	0.20 (0.09,0.66)	0.39 (0.16,0.83)	0.064
K_ep_min_ (min^−1^)	0.01 (0.00,0.06)	0.000 (0.00,0.12)	0.571
K_ep_max_ (min^−1^)	0.75 (0.46,0.97)	1.80 (1.15,3.07)	**<0.001**
K_ep_median_ (min^−1^)	0.28 (0.19,0.49)	0.52 (0.37,0.75)	**0.001**
K_ep_mean_ (min^−1^)	0.31 (0.19,0.50)	0.61 (0.39,0.82)	**<0.001**
V_e_min_	0.11 (0.00,0.32)	0.00 (0.00,0.28)	0.222
V_e_max_	1.00 (1.00,1.00)	1.00 (1.00,1.00)	0.964
V_e_median_	0.75 (0.38,1.00)	0.68 (0.33,0.98)	0.632
V_e_mean_	0.73 (0.41,0.93)	0.68 (0.38,0.87)	0.477

Normally distributed data are presented as mean±standard deviation, and non-normal data are presented as median (interquartile range). The *p* value was calculated with the two independent samples *t*-test (^#^) or a Mann–Whitney U test. *p* values < 0.05 are presented in bold.

**Table 4:** Diagnostic performance of the models.

Model	Variables	AUC	Standard Error	95% Confidence Interval		Accuracy (%)	Sensitivity (%)	Specificity (%)
				Lower Bound	Upper Bound			
model__ADC_	ADC__min_	0.768	0.063	0.672	0.846	0.789	75.00	75.00
model__IVIM_	D__mean_	0.826	0.044	0.737	0.894	0.820	72.50	80.00
model__DCE_	K_ep_max_	0.823	0.056	0.734	0.892	0.793	78.75	85.00
model__DCE+ADC_	K_ep_max_, ADC__min_	0.852	0.049	0.768	0.915	0.789	86.25	75.00
model__DCE+IVIM_	K_ep_max_, D__mean_	0.903	0.037	0.828	0.953	0.789	87.50	85.00

**Table 5:** Models with significant difference in ROC curves after the Delong test.

Model	Standard Error	*p* Value
model__DCE+IVIM_ vs. model__IVIM_	0.036	0.033
model__DCE+IVIM_ vs. model__ADC_	0.055	0.014

ROC, receiver operating characteristic.
